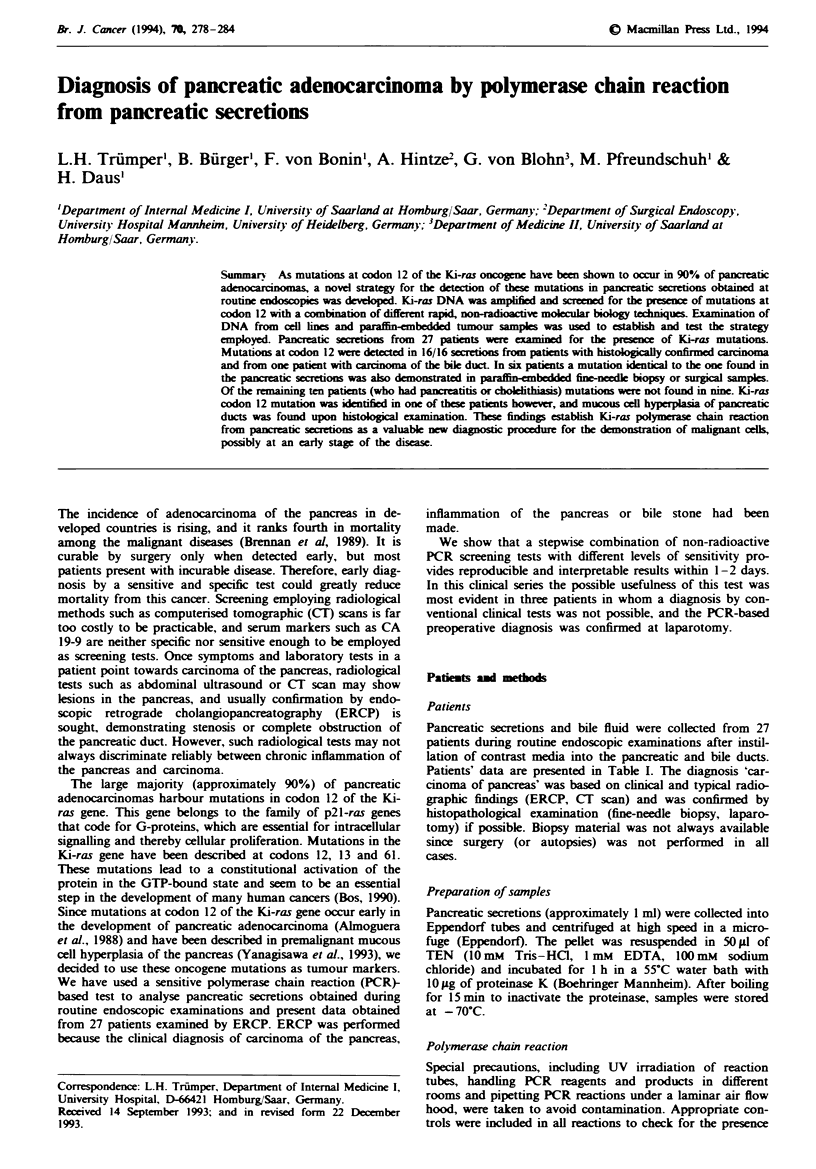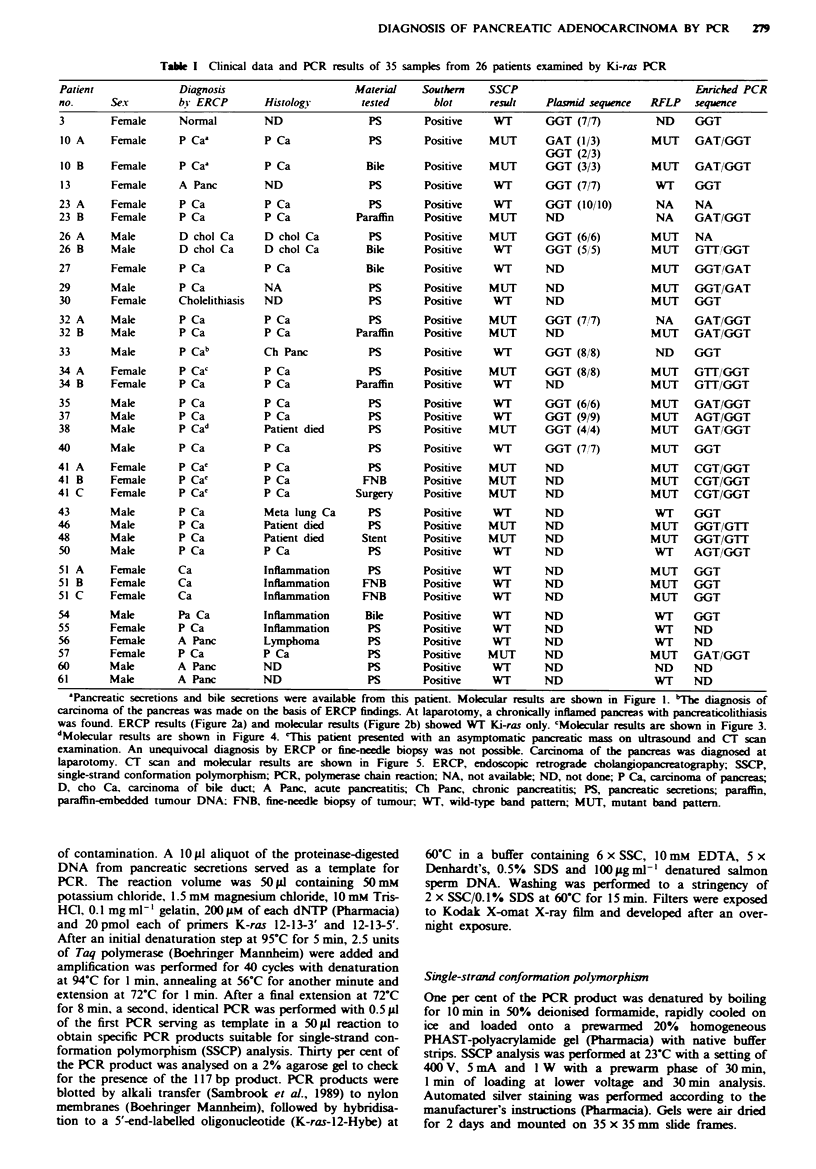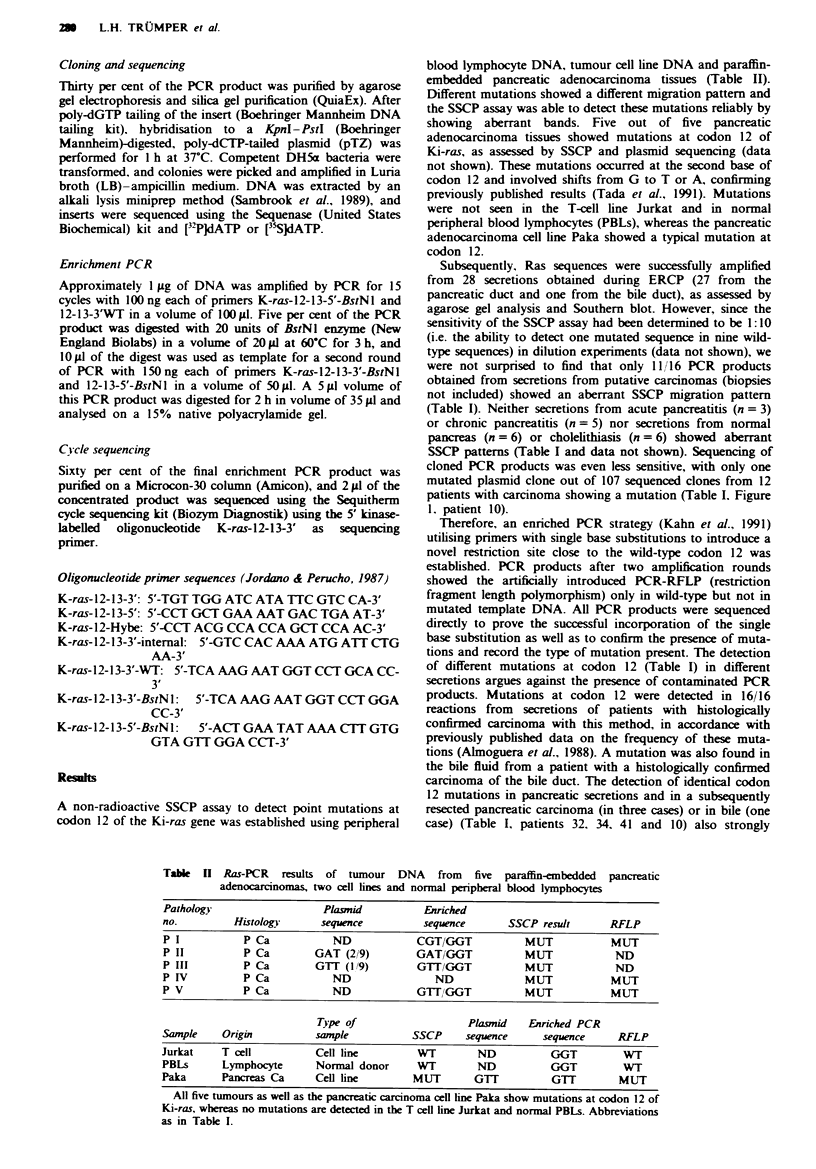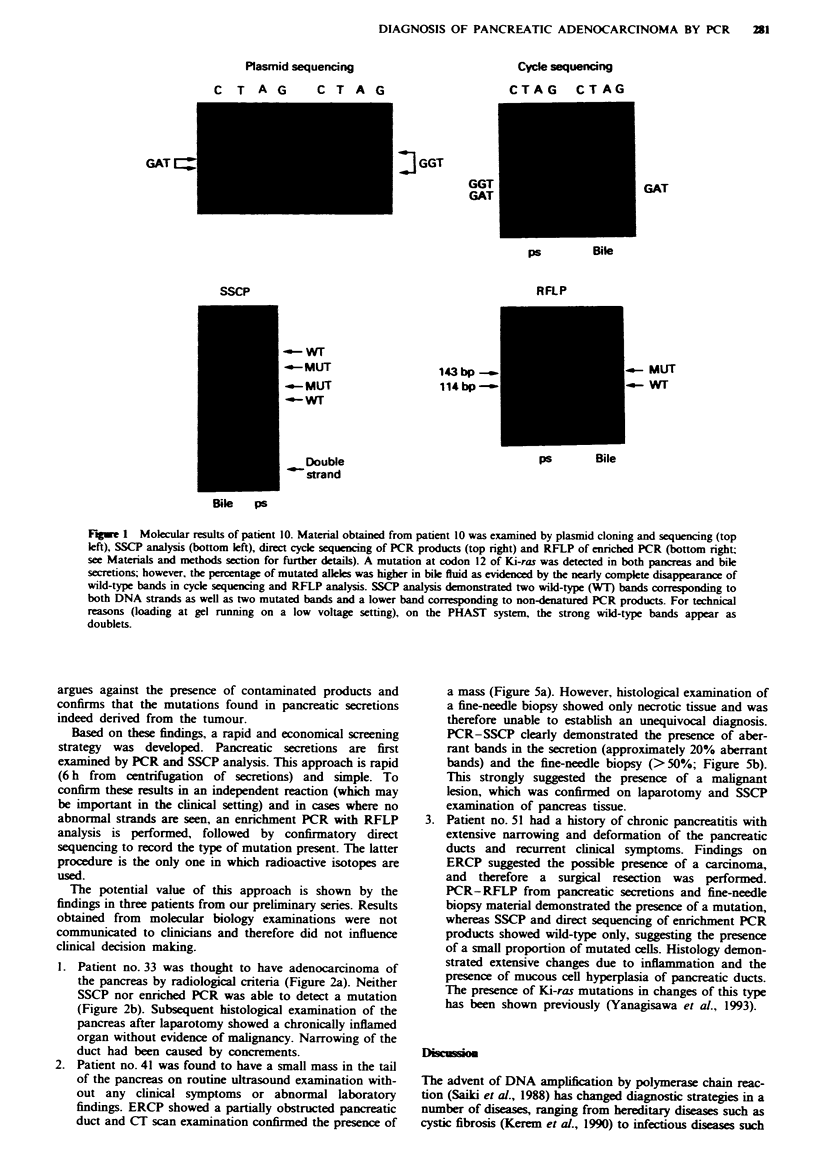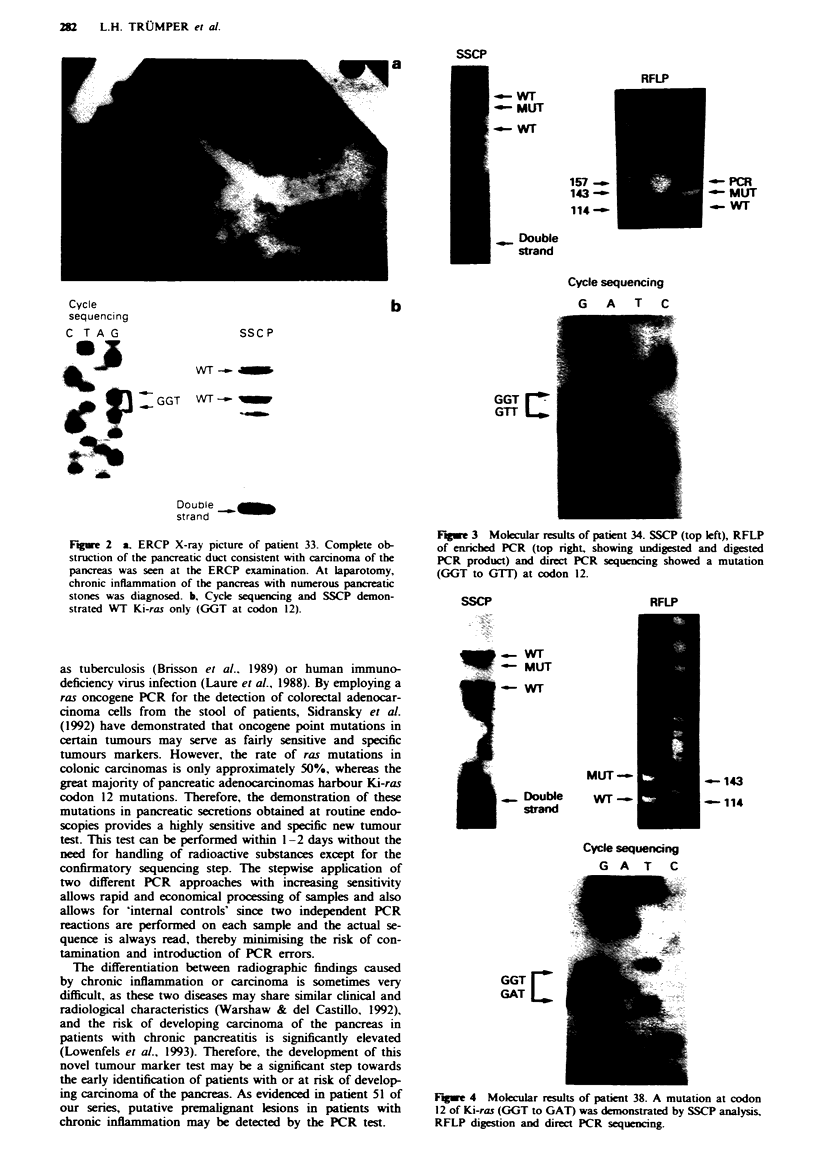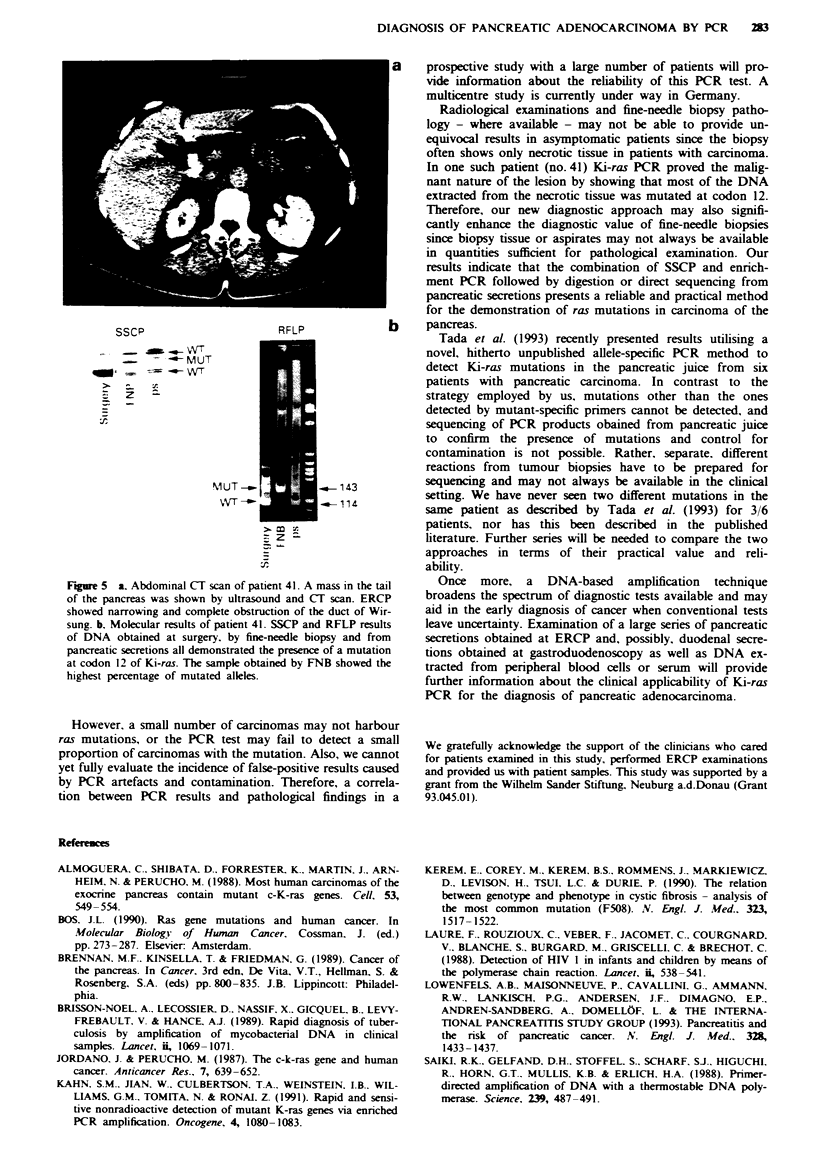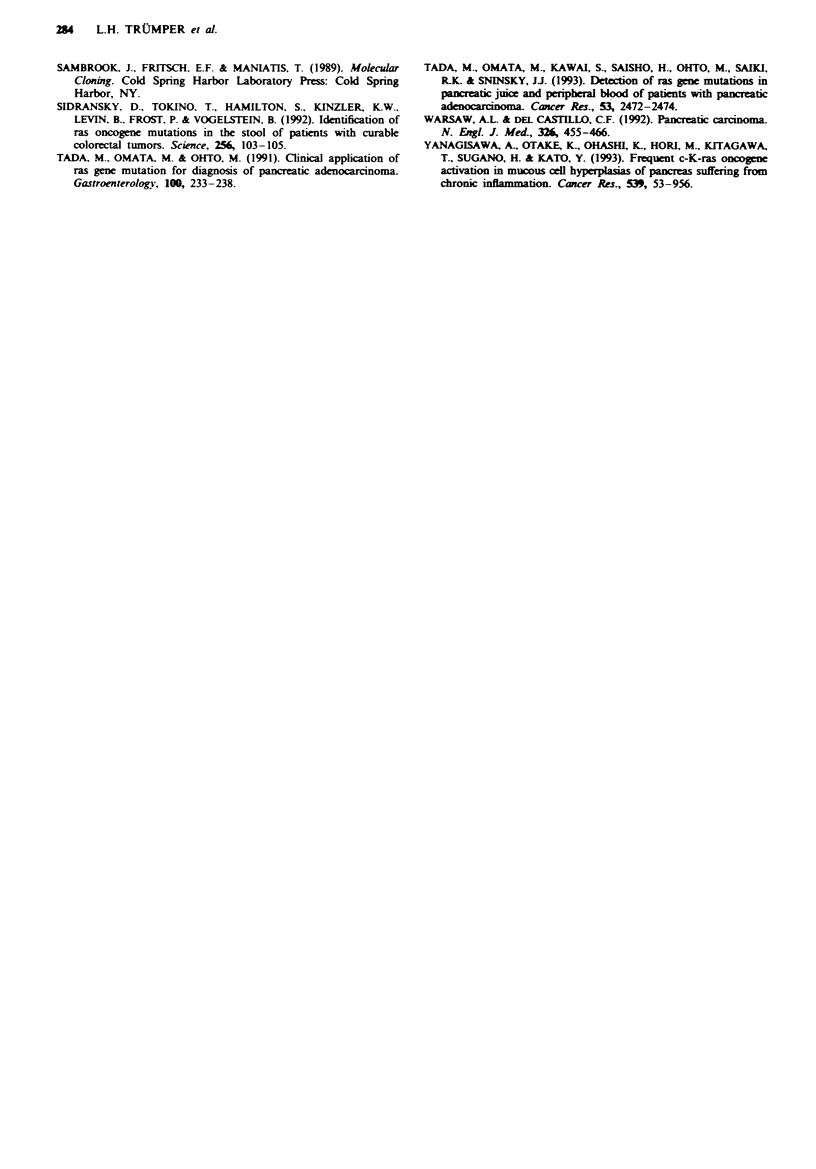# Diagnosis of pancreatic adenocarcinoma by polymerase chain reaction from pancreatic secretions.

**DOI:** 10.1038/bjc.1994.292

**Published:** 1994-08

**Authors:** L. H. Trümper, B. Bürger, F. von Bonin, A. Hintze, G. von Blohn, M. Pfreundschuh, H. Daus

**Affiliations:** Department of Internal Medicine I, University of Saarland at Homburg/Saar, Germany.

## Abstract

**Images:**


Br. J. Cancer (1994), 70, 278-284 © Macmillan Press Ltd., 1994

### Diagnosis of pancreatic adenocarcinoma by polymerase chain reaction from pancreatic secretions

L.H. Trümper[1], B. Bürger[1], F. von Bonin[1], A. Hintze[2], G. von Blohn[3], M. Pfreundschuh[1] & H. Daus[1]

[1]*Department of Internal Medicine I, University of Saarland at Homburg/Saar, Germany;* [2]*Department of Surgical Endoscopy, University Hospital Mannheim, University of Heidelberg, Germany;* [3]*Department of Medicine II, University of Saarland at Homburg/Saar, Germany.*

Summary As mutations at codon 12 of the Ki-*ras* oncogene have been shown to occur in 90% of pancreatic adenocarcinomas, a novel strategy for the detection of these mutations in pancreatic secretions obtained at routine endoscopies was developed. Ki-*ras* DNA was amplified and screened for the presence of mutations at codon 12 with a combination of different rapid, non-radioactive molecular biology techniques. Examination of DNA from cell lines and paraffin-embedded tumour samples was used to establish and test the strategy employed. Pancreatic secretions from 27 patients were examined for the presence of Ki-*ras* mutations. Mutations at codon 12 were detected in 16/16 secretions from patients with histologically confirmed carcinoma and from one patient with carcinoma of the bile duct. In six patients a mutation identical to the one found in the pancreatic secretions was also demonstrated in paraffin-embedded fine-needle biopsy or surgical samples. Of the remaining ten patients (who had pancreatitis or cholelithiasis) mutations were not found in nine. Ki-*ras* codon 12 mutation was identified in one of these patients however, and mucous cell hyperplasia of pancreatic ducts was found upon histological examination. These findings establish Ki-*ras* polymerase chain reaction from pancreatic secretions as a valuable new diagnostic procedure for the demonstration of malignant cells, possibly at an early stage of the disease.

The incidence of adenocarcinoma of the pancreas in developed countries is rising, and it ranks fourth in mortality among the malignant diseases (Brennan *et al*, 1989). It is curable by surgery only when detected early, but most patients present with incurable disease. Therefore, early diagnosis by a sensitive and specific test could greatly reduce mortality from this cancer. Screening employing radiological methods such as computerised tomographic (CT) scans is far too costly to be practicable, and serum markers such as CA 19-9 are neither specific nor sensitive enough to be employed as screening tests. Once symptoms and laboratory tests in a patient point towards carcinoma of the pancreas, radiological tests such as abdominal ultrasound or CT scan may show lesions in the pancreas, and usually confirmation by endoscopic retrograde cholangiopancreatography (ERCP) is sought, demonstrating stenosis or complete obstruction of the pancreatic duct. However, such radiological tests may not always discriminate reliably between chronic inflammation of the pancreas and carcinoma.

The large majority (approximately 90%) of pancreatic adenocarcinomas harbour mutations in codon 12 of the Ki-*ras* gene. This gene belongs to the family of p21-*ras* genes that code for G-proteins, which are essential for intracellular signalling and thereby cellular proliferation. Mutations in the Ki-*ras* gene have been described at codons 12, 13 and 61. These mutations lead to a constitutional activation of the protein in the GTP-bound state and seem to be an essential step in the development of many human cancers (Bos, 1990). Since mutations at codon 12 of the Ki-*ras* gene occur early in the development of pancreatic adenocarcinoma (Almoguera *et al*., 1988) and have been described in premalignant mucous cell hyperplasia of the pancreas (Yanagisawa *et al*., 1993), we decided to use these oncogene mutations as tumour markers. We have used a sensitive polymerase chain reaction (PCR)-based test to analyse pancreatic secretions obtained during routine endoscopic examinations and present data obtained from 27 patients examined by ERCP. ERCP was performed because the clinical diagnosis of carcinoma of the pancreas, inflammation of the pancreas or bile stone had been made.

We show that a stepwise combination of non-radioactive PCR screening tests with different levels of sensitivity provides reproducible and interpretable results within 1-2 days. In this clinical series the possible usefulness of this test was most evident in three patients in whom a diagnosis by conventional clinical tests was not possible, and the PCR-based preoperative diagnosis was confirmed at laparotomy.

### Patients and methods

### *Patients*

Pancreatic secretions and bile fluid were collected from 27 patients during routine endoscopic examinations after instillation of contrast media into the pancreatic and bile ducts. Patients' data are presented in Table I. The diagnosis 'carcinoma of pancreas' was based on clinical and typical radiographic findings (ERCP, CT scan) and was confirmed by histopathological examination (fine-needle biopsy, laparotomy) if possible. Biopsy material was not always available since surgery (or autopsies) was not performed in all cases.

### *Preparation of samples*

Pancreatic secretions (approximately 1 ml) were collected into Eppendorf tubes and centrifuged at high speed in a microfuge (Eppendorf). The pellet was resuspended in 50 µl of TEN (10 mM Tris-HCl, 1 mM EDTA, 100 mM sodium chloride) and incubated for 1 h in a 55°C water bath with 10 µg of proteinase K (Boehringer Mannheim). After boiling for 15 min to inactivate the proteinase, samples were stored at −70°C.

### *Polymerase chain reaction*

Special precautions, including UV irradiation of reaction tubes, handling PCR reagents and products in different rooms and pipetting PCR reactions under a laminar air flow hood, were taken to avoid contamination. Appropriate controls were included in all reactions to check for the presence

Correspondence: L.H. Trümper, Department of Internal Medicine I, University Hospital, D-66421 Homburg/Saar, Germany.
Received 14 September 1993; and in revised form 22 December 1993.

**Table I** Clinical data and PCR results of 35 samples from 26 patients examined by Ki-*ras* PCR

| Patient no. | Sex | Diagnosis by ERCP | Histology | Material tested | Southern blot | SSCP result | Plasmid sequence | RFLP | Enriched PCR sequence |
|---|---|---|---|---|---|---|---|---|---|
| 3 | Female | Normal | ND | PS | Positive | WT | GGT (7/7) | ND | GGT |
| 10 A | Female | P Ca[a] | P Ca | PS | Positive | MUT | GAT (1/3) GGT (2/3) | MUT | GAT/GGT |
| 10 B | Female | P Ca[a] | P Ca | Bile | Positive | MUT | GGT (3/3) | MUT | GAT/GGT |
| 13 | Female | A Panc | ND | PS | Positive | WT | GGT (7/7) | WT | GGT |
| 23 A | Female | P Ca | P Ca | PS | Positive | WT | GGT (10/10) | NA | NA |
| 23 B | Female | P Ca | P Ca | Paraffin | Positive | MUT | ND | NA | GAT/GGT |
| 26 A | Male | D chol Ca | D chol Ca | PS | Positive | MUT | GGT (6/6) | MUT | NA |
| 26 B | Male | D chol Ca | D chol Ca | Bile | Positive | WT | GGT (5/5) | MUT | GTT/GGT |
| 27 | Female | P Ca | P Ca | Bile | Positive | WT | ND | MUT | GGT/GAT |
| 29 | Male | P Ca | NA | PS | Positive | MUT | ND | MUT | GGT/GAT |
| 30 | Female | Cholelithiasis | ND | PS | Positive | WT | ND | MUT | GGT |
| 32 A | Male | P Ca | P Ca | PS | Positive | MUT | GGT (7/7) | NA | GAT/GGT |
| 32 B | Male | P Ca | P Ca | Paraffin | Positive | MUT | ND | MUT | GAT/GGT |
| 33 | Male | P Ca[b] | Ch Panc | PS | Positive | WT | GGT (8/8) | ND | GGT |
| 34 A | Female | P Ca[c] | P Ca | PS | Positive | MUT | GGT (8/8) | MUT | GTT/GGT |
| 34 B | Female | P Ca | P Ca | Paraffin | Positive | WT | ND | MUT | GTT/GGT |
| 35 | Male | P Ca | P Ca | PS | Positive | WT | GGT (6/6) | MUT | GAT/GGT |
| 37 | Male | P Ca | P Ca | PS | Positive | WT | GGT (9/9) | MUT | AGT/GGT |
| 38 | Male | P Ca[d] | Patient died | PS | Positive | MUT | GGT (4/4) | MUT | GAT/GGT |
| 40 | Male | P Ca | P Ca | PS | Positive | WT | GGT (7/7) | MUT | GGT |
| 41 A | Female | P Ca[e] | P Ca | PS | Positive | MUT | ND | MUT | CGT/GGT |
| 41 B | Female | P Ca[e] | P Ca | FNB | Positive | MUT | ND | MUT | CGT/GGT |
| 41 C | Female | P Ca[e] | P Ca | Surgery | Positive | MUT | ND | MUT | CGT/GGT |
| 43 | Male | P Ca | Meta lung Ca | PS | Positive | WT | ND | WT | GGT |
| 46 | Male | P Ca | Patient died | PS | Positive | MUT | ND | MUT | GGT/GTT |
| 48 | Male | P Ca | Patient died | Stent | Positive | MUT | ND | MUT | GGT/GTT |
| 50 | Male | P Ca | P Ca | PS | Positive | WT | ND | WT | AGT/GGT |
| 51 A | Female | Ca | Inflammation | PS | Positive | WT | ND | MUT | GGT |
| 51 B | Female | Ca | Inflammation | FNB | Positive | WT | ND | MUT | GGT |
| 51 C | Female | Ca | Inflammation | FNB | Positive | WT | ND | MUT | GGT |
| 54 | Male | Pa Ca | Inflammation | Bile | Positive | WT | ND | WT | GGT |
| 55 | Female | P Ca | Inflammation | PS | Positive | WT | ND | WT | ND |
| 56 | Female | A Panc | Lymphoma | PS | Positive | WT | ND | WT | ND |
| 57 | Female | P Ca | P Ca | PS | Positive | MUT | ND | MUT | GAT/GGT |
| 60 | Male | A Panc | ND | PS | Positive | WT | ND | ND | ND |
| 61 | Male | A Panc | ND | PS | Positive | WT | ND | WT | ND |

[a]Pancreatic secretions and bile secretions were available from this patient. Molecular results are shown in Figure 1. [b]The diagnosis of carcinoma of the pancreas was made on the basis of ERCP findings. At laparotomy, a chronically inflamed pancreas with pancreaticolithiasis was found. ERCP results (Figure 2a) and molecular results (Figure 2b) showed WT Ki-*ras* only. [c]Molecular results are shown in Figure 3. [d]Molecular results are shown in Figure 4. [e]This patient presented with an asymptomatic pancreatic mass on ultrasound and CT scan examination. An unequivocal diagnosis by ERCP or fine-needle biopsy was not possible. Carcinoma of the pancreas was diagnosed at laparotomy. CT scan and molecular results are shown in Figure 5. ERCP, endoscopic retrograde cholangiopancreatography; SSCP, single-strand conformation polymorphism; PCR, polymerase chain reaction; NA, not available; ND, not done; P Ca, carcinoma of pancreas; D, cho Ca, carcinoma of bile duct; A Panc, acute pancreatitis; Ch Panc, chronic pancreatitis; PS, pancreatic secretions; paraffin, paraffin-embedded tumour DNA; FNB, fine-needle biopsy of tumour; WT, wild-type band pattern; MUT, mutant band pattern.

of contamination. A 10 μl aliquot of the proteinase-digested DNA from pancreatic secretions served as a template for PCR. The reaction volume was 50 μl containing 50 mM potassium chloride, 1.5 mM magnesium chloride, 10 mM Tris-HCl, 0.1 mg $ml^{-1}$ gelatin, 200 μM of each dNTP (Pharmacia) and 20 pmol each of primers K-*ras* 12-13-3′ and 12-13-5′. After an initial denaturation step at 95°C for 5 min, 2.5 units of *Taq* polymerase (Boehringer Mannheim) were added and amplification was performed for 40 cycles with denaturation at 94°C for 1 min, annealing at 56°C for another minute and extension at 72°C for 1 min. After a final extension at 72°C for 8 min, a second, identical PCR was performed with 0.5 μl of the first PCR serving as template in a 50 μl reaction to obtain specific PCR products suitable for single-strand conformation polymorphism (SSCP) analysis. Thirty per cent of the PCR product was analysed on a 2% agarose gel to check for the presence of the 117 bp product. PCR products were blotted by alkali transfer (Sambrook *et al.*, 1989) to nylon membranes (Boehringer Mannheim), followed by hybridisation to a 5′-end-labelled oligonucleotide (K-*ras*-12-Hybe) at 60°C in a buffer containing 6 × SSC, 10 mM EDTA, 5 × Denhardt's, 0.5% SDS and 100 μg $ml^{-1}$ denatured salmon sperm DNA. Washing was performed to a stringency of 2 × SSC/0.1% SDS at 60°C for 15 min. Filters were exposed to Kodak X-omat X-ray film and developed after an overnight exposure.

### *Single-strand conformation polymorphism*

One per cent of the PCR product was denatured by boiling for 10 min in 50% deionised formamide, rapidly cooled on ice and loaded onto a prewarmed 20% homogeneous PHAST-polyacrylamide gel (Pharmacia) with native buffer strips. SSCP analysis was performed at 23°C with a setting of 400 V, 5 mA and 1 W with a prewarm phase of 30 min, 1 min of loading at lower voltage and 30 min analysis. Automated silver staining was performed according to the manufacturer's instructions (Pharmacia). Gels were air dried for 2 days and mounted on 35 × 35 mm slide frames.

*Cloning and sequencing*

Thirty per cent of the PCR product was purified by agarose gel electrophoresis and silica gel purification (QuiaEx). After poly-dGTP tailing of the insert (Boehringer Mannheim DNA tailing kit), hybridisation to a *Kpn*I–*Pst*I (Boehringer Mannheim)-digested, poly-dCTP-tailed plasmid (pTZ) was performed for 1 h at 37°C. Competent DH5α bacteria were transformed, and colonies were picked and amplified in Luria broth (LB)–ampicillin medium. DNA was extracted by an alkali lysis miniprep method (Sambrook *et al.*, 1989), and inserts were sequenced using the Sequenase (United States Biochemical) kit and [$^{32}$P]dATP or [$^{35}$S]dATP.

*Enrichment PCR*

Approximately 1 µg of DNA was amplified by PCR for 15 cycles with 100 ng each of primers K-*ras*-12-13-5′-*Bst*N1 and 12-13-3′WT in a volume of 100 µl. Five per cent of the PCR product was digested with 20 units of *Bst*N1 enzyme (New England Biolabs) in a volume of 20 µl at 60°C for 3 h, and 10 µl of the digest was used as template for a second round of PCR with 150 ng each of primers K-*ras*-12-13-3′-*Bst*N1 and 12-13-5′-*Bst*N1 in a volume of 50 µl. A 5 µl volume of this PCR product was digested for 2 h in volume of 35 µl and analysed on a 15% native polyacrylamide gel.

*Cycle sequencing*

Sixty per cent of the final enrichment PCR product was purified on a Microcon-30 column (Amicon), and 2 µl of the concentrated product was sequenced using the Sequitherm cycle sequencing kit (Biozym Diagnostik) using the 5′ kinase-labelled oligonucleotide K-*ras*-12-13-3′ as sequencing primer.

*Oligonucleotide primer sequences (Jordano & Perucho, 1987)*

K-*ras*-12-13-3′: 5′-TGT TGG ATC ATA TTC GTC CA-3′
K-*ras*-12-13-5′: 5′-CCT GCT GAA AAT GAC TGA AT-3′
K-*ras*-12-Hybe: 5′-CCT ACG CCA CCA GCT CCA AC-3′
K-*ras*-12-13-3′-internal: 5′-GTC CAC AAA ATG ATT CTG AA-3′
K-*ras*-12-13-3′-WT: 5′-TCA AAG AAT GGT CCT GCA CC-3′
K-*ras*-12-13-3′-*Bst*N1: 5′-TCA AAG AAT GGT CCT GGA CC-3′
K-*ras*-12-13-5′-*Bst*N1: 5′-ACT GAA TAT AAA CTT GTG GTA GTT GGA CCT-3′

### Results

A non-radioactive SSCP assay to detect point mutations at codon 12 of the Ki-*ras* gene was established using peripheral blood lymphocyte DNA, tumour cell line DNA and paraffin-embedded pancreatic adenocarcinoma tissues (Table II). Different mutations showed a different migration pattern and the SSCP assay was able to detect these mutations reliably by showing aberrant bands. Five out of five pancreatic adenocarcinoma tissues showed mutations at codon 12 of Ki-*ras*, as assessed by SSCP and plasmid sequencing (data not shown). These mutations occurred at the second base of codon 12 and involved shifts from G to T or A, confirming previously published results (Tada *et al.*, 1991). Mutations were not seen in the T-cell line Jurkat and in normal peripheral blood lymphocytes (PBLs), whereas the pancreatic adenocarcinoma cell line Paka showed a typical mutation at codon 12.

Subsequently, Ras sequences were successfully amplified from 28 secretions obtained during ERCP (27 from the pancreatic duct and one from the bile duct), as assessed by agarose gel analysis and Southern blot. However, since the sensitivity of the SSCP assay had been determined to be 1:10 (i.e. the ability to detect one mutated sequence in nine wild-type sequences) in dilution experiments (data not shown), we were not surprised to find that only 11/16 PCR products obtained from secretions from putative carcinomas (biopsies not included) showed an aberrant SSCP migration pattern (Table I). Neither secretions from acute pancreatitis ($n = 3$) or chronic pancreatitis ($n = 5$) nor secretions from normal pancreas ($n = 6$) or cholelithiasis ($n = 6$) showed aberrant SSCP patterns (Table I and data not shown). Sequencing of cloned PCR products was even less sensitive, with only one mutated plasmid clone out of 107 sequenced clones from 12 patients with carcinoma showing a mutation (Table I, Figure 1, patient 10).

Therefore, an enriched PCR strategy (Kahn *et al.*, 1991) utilising primers with single base substitutions to introduce a novel restriction site close to the wild-type codon 12 was established. PCR products after two amplification rounds showed the artificially introduced PCR-RFLP (restriction fragment length polymorphism) only in wild-type but not in mutated template DNA. All PCR products were sequenced directly to prove the successful incorporation of the single base substitution as well as to confirm the presence of mutations and record the type of mutation present. The detection of different mutations at codon 12 (Table I) in different secretions argues against the presence of contaminated PCR products. Mutations at codon 12 were detected in 16/16 reactions from secretions of patients with histologically confirmed carcinoma with this method, in accordance with previously published data on the frequency of these mutations (Almoguera *et al.*, 1988). A mutation was also found in the bile fluid from a patient with a histologically confirmed carcinoma of the bile duct. The detection of identical codon 12 mutations in pancreatic secretions and in a subsequently resected pancreatic carcinoma (in three cases) or in bile (one case) (Table I, patients 32, 34, 41 and 10) also strongly

**Table II** *Ras*-PCR results of tumour DNA from five paraffin-embedded pancreatic adenocarcinomas, two cell lines and normal peripheral blood lymphocytes

| *Pathology no.* | *Histology* | *Plasmid sequence* | *Enriched sequence* | *SSCP result* | *RFLP* |
|---|---|---|---|---|---|
| P I | P Ca | ND | CGT/GGT | MUT | MUT |
| P II | P Ca | GAT (2/9) | GAT/GGT | MUT | ND |
| P III | P Ca | GTT (1/9) | GTT/GGT | MUT | ND |
| P IV | P Ca | ND | ND | MUT | MUT |
| P V | P Ca | ND | GTT/GGT | MUT | MUT |

| *Sample* | *Origin* | *Type of sample* | *SSCP* | *Plasmid sequence* | *Enriched PCR sequence* | *RFLP* |
|---|---|---|---|---|---|---|
| Jurkat | T cell | Cell line | WT | ND | GGT | WT |
| PBLs | Lymphocyte | Normal donor | WT | ND | GGT | WT |
| Paka | Pancreas Ca | Cell line | MUT | GTT | GTT | MUT |

All five tumours as well as the pancreatic carcinoma cell line Paka show mutations at codon 12 of Ki-*ras*, whereas no mutations are detected in the T cell line Jurkat and normal PBLs. Abbreviations as in Table I.

**Figure 1** Molecular results of patient 10. Material obtained from patient 10 was examined by plasmid cloning and sequencing (top left), SSCP analysis (bottom left), direct cycle sequencing of PCR products (top right) and RFLP of enriched PCR (bottom right; see Materials and methods section for further details). A mutation at codon 12 of Ki-*ras* was detected in both pancreas and bile secretions; however, the percentage of mutated alleles was higher in bile fluid as evidenced by the nearly complete disappearance of wild-type bands in cycle sequencing and RFLP analysis. SSCP analysis demonstrated two wild-type (WT) bands corresponding to both DNA strands as well as two mutated bands and a lower band corresponding to non-denatured PCR products. For technical reasons (loading at gel running on a low voltage setting), on the PHAST system, the strong wild-type bands appear as doublets.

argues against the presence of contaminated products and confirms that the mutations found in pancreatic secretions indeed derived from the tumour.

Based on these findings, a rapid and economical screening strategy was developed. Pancreatic secretions are first examined by PCR and SSCP analysis. This approach is rapid (6 h from centrifugation of secretions) and simple. To confirm these results in an independent reaction (which may be important in the clinical setting) and in cases where no abnormal strands are seen, an enrichment PCR with RFLP analysis is performed, followed by confirmatory direct sequencing to record the type of mutation present. The latter procedure is the only one in which radioactive isotopes are used.

The potential value of this approach is shown by the findings in three patients from our preliminary series. Results obtained from molecular biology examinations were not communicated to clinicians and therefore did not influence clinical decision making.

1. Patient no. 33 was thought to have adenocarcinoma of the pancreas by radiological criteria (Figure 2a). Neither SSCP nor enriched PCR was able to detect a mutation (Figure 2b). Subsequent histological examination of the pancreas after laparotomy showed a chronically inflamed organ without evidence of malignancy. Narrowing of the duct had been caused by concrements.
2. Patient no. 41 was found to have a small mass in the tail of the pancreas on routine ultrasound examination without any clinical symptoms or abnormal laboratory findings. ERCP showed a partially obstructed pancreatic duct and CT scan examination confirmed the presence of a mass (Figure 5a). However, histological examination of a fine-needle biopsy showed only necrotic tissue and was therefore unable to establish an unequivocal diagnosis. PCR–SSCP clearly demonstrated the presence of aberrant bands in the secretion (approximately 20% aberrant bands) and the fine-needle biopsy (>50%; Figure 5b). This strongly suggested the presence of a malignant lesion, which was confirmed on laparotomy and SSCP examination of pancreas tissue.
3. Patient no. 51 had a history of chronic pancreatitis with extensive narrowing and deformation of the pancreatic ducts and recurrent clinical symptoms. Findings on ERCP suggested the possible presence of a carcinoma, and therefore a surgical resection was performed. PCR–RFLP from pancreatic secretions and fine-needle biopsy material demonstrated the presence of a mutation, whereas SSCP and direct sequencing of enrichment PCR products showed wild-type only, suggesting the presence of a small proportion of mutated cells. Histology demonstrated extensive changes due to inflammation and the presence of mucous cell hyperplasia of pancreatic ducts. The presence of Ki-*ras* mutations in changes of this type has been shown previously (Yanagisawa *et al.*, 1993).

### Discussion

The advent of DNA amplification by polymerase chain reaction (Saiki *et al.*, 1988) has changed diagnostic strategies in a number of diseases, ranging from hereditary diseases such as cystic fibrosis (Kerem *et al.*, 1990) to infectious diseases such

**Figure 2** **a**, ERCP X-ray picture of patient 33. Complete obstruction of the pancreatic duct consistent with carcinoma of the pancreas was seen at the ERCP examination. At laparotomy, chronic inflammation of the pancreas with numerous pancreatic stones was diagnosed. **b**, Cycle sequencing and SSCP demonstrated WT Ki-*ras* only (GGT at codon 12).

**Figure 3** Molecular results of patient 34. SSCP (top left), RFLP of enriched PCR (top right, showing undigested and digested PCR product) and direct PCR sequencing showed a mutation (GGT to GTT) at codon 12.

**Figure 4** Molecular results of patient 38. A mutation at codon 12 of Ki-*ras* (GGT to GAT) was demonstrated by SSCP analysis, RFLP digestion and direct PCR sequencing.

as tuberculosis (Brisson *et al.*, 1989) or human immunodeficiency virus infection (Laure *et al.*, 1988). By employing a *ras* oncogene PCR for the detection of colorectal adenocarcinoma cells from the stool of patients, Sidransky *et al.* (1992) have demonstrated that oncogene point mutations in certain tumours may serve as fairly sensitive and specific tumours markers. However, the rate of *ras* mutations in colonic carcinomas is only approximately 50%, whereas the great majority of pancreatic adenocarcinomas harbour Ki-*ras* codon 12 mutations. Therefore, the demonstration of these mutations in pancreatic secretions obtained at routine endoscopies provides a highly sensitive and specific new tumour test. This test can be performed within 1–2 days without the need for handling of radioactive substances except for the confirmatory sequencing step. The stepwise application of two different PCR approaches with increasing sensitivity allows rapid and economical processing of samples and also allows for 'internal controls' since two independent PCR reactions are performed on each sample and the actual sequence is always read, thereby minimising the risk of contamination and introduction of PCR errors.

The differentiation between radiographic findings caused by chronic inflammation or carcinoma is sometimes very difficult, as these two diseases may share similar clinical and radiological characteristics (Warshaw & del Castillo, 1992), and the risk of developing carcinoma of the pancreas in patients with chronic pancreatitis is significantly elevated (Lowenfels *et al.*, 1993). Therefore, the development of this novel tumour marker test may be a significant step towards the early identification of patients with or at risk of developing carcinoma of the pancreas. As evidenced in patient 51 of our series, putative premalignant lesions in patients with chronic inflammation may be detected by the PCR test.

**Figure 5** **a**, Abdominal CT scan of patient 41. A mass in the tail of the pancreas was shown by ultrasound and CT scan. ERCP showed narrowing and complete obstruction of the duct of Wirsung. **b**, Molecular results of patient 41. SSCP and RFLP results of DNA obtained at surgery, by fine-needle biopsy and from pancreatic secretions all demonstrated the presence of a mutation at codon 12 of Ki-*ras*. The sample obtained by FNB showed the highest percentage of mutated alleles.

However, a small number of carcinomas may not harbour *ras* mutations, or the PCR test may fail to detect a small proportion of carcinomas with the mutation. Also, we cannot yet fully evaluate the incidence of false-positive results caused by PCR artefacts and contamination. Therefore, a correlation between PCR results and pathological findings in a prospective study with a large number of patients will provide information about the reliability of this PCR test. A multicentre study is currently under way in Germany.

Radiological examinations and fine-needle biopsy pathology – where available – may not be able to provide unequivocal results in asymptomatic patients since the biopsy often shows only necrotic tissue in patients with carcinoma. In one such patient (no. 41) Ki-*ras* PCR proved the malignant nature of the lesion by showing that most of the DNA extracted from the necrotic tissue was mutated at codon 12. Therefore, our new diagnostic approach may also significantly enhance the diagnostic value of fine-needle biopsies since biopsy tissue or aspirates may not always be available in quantities sufficient for pathological examination. Our results indicate that the combination of SSCP and enrichment PCR followed by digestion or direct sequencing from pancreatic secretions presents a reliable and practical method for the demonstration of *ras* mutations in carcinoma of the pancreas.

Tada *et al.* (1993) recently presented results utilising a novel, hitherto unpublished allele-specific PCR method to detect Ki-*ras* mutations in the pancreatic juice from six patients with pancreatic carcinoma. In contrast to the strategy employed by us, mutations other than the ones detected by mutant-specific primers cannot be detected, and sequencing of PCR products obained from pancreatic juice to confirm the presence of mutations and control for contamination is not possible. Rather, separate, different reactions from tumour biopsies have to be prepared for sequencing and may not always be available in the clinical setting. We have never seen two different mutations in the same patient as described by Tada *et al.* (1993) for 3/6 patients, nor has this been described in the published literature. Further series will be needed to compare the two approaches in terms of their practical value and reliability.

Once more, a DNA-based amplification technique broadens the spectrum of diagnostic tests available and may aid in the early diagnosis of cancer when conventional tests leave uncertainty. Examination of a large series of pancreatic secretions obtained at ERCP and, possibly, duodenal secretions obtained at gastroduodenoscopy as well as DNA extracted from peripheral blood cells or serum will provide further information about the clinical applicability of Ki-*ras* PCR for the diagnosis of pancreatic adenocarcinoma.

We gratefully acknowledge the support of the clinicians who cared for patients examined in this study, performed ERCP examinations and provided us with patient samples. This study was supported by a grant from the Wilhelm Sander Stiftung, Neuburg a.d.Donau (Grant 93.045.01).

### References

ALMOGUERA, C., SHIBATA, D., FORRESTER, K., MARTIN, J., ARNHEIM, N. & PERUCHO, M. (1988). Most human carcinomas of the exocrine pancreas contain mutant c-K-ras genes. *Cell*, **53**, 549–554.

BOS, J.L. (1990). Ras gene mutations and human cancer. In *Molecular Biology of Human Cancer*, Cossman, J. (ed.) pp. 273–287. Elsevier: Amsterdam.

BRENNAN, M.F., KINSELLA, T. & FRIEDMAN, G. (1989). Cancer of the pancreas. In *Cancer*, 3rd edn, De Vita, V.T., Hellman, S. & Rosenberg, S.A. (eds) pp. 800–835. J.B. Lippincott: Philadelphia.

BRISSON-NOEL, A., LECOSSIER, D., NASSIF, X., GICQUEL, B., LEVY-FREBAULT, V. & HANCE, A.J. (1989). Rapid diagnosis of tuberculosis by amplification of mycobacterial DNA in clinical samples. *Lancet*, **ii**, 1069–1071.

JORDANO, J. & PERUCHO, M. (1987). The c-k-ras gene and human cancer. *Anticancer Res.*, **7**, 639–652.

KAHN, S.M., JIAN, W., CULBERTSON, T.A., WEINSTEIN, I.B., WILLIAMS, G.M., TOMITA, N. & RONAI, Z. (1991). Rapid and sensitive nonradioactive detection of mutant K-ras genes via enriched PCR amplification. *Oncogene*, **4**, 1080–1083.

KEREM, E., COREY, M., KEREM, B.S., ROMMENS, J., MARKIEWICZ, D., LEVISON, H., TSUI, L.C. & DURIE, P. (1990). The relation between genotype and phenotype in cystic fibrosis – analysis of the most common mutation (F508). *N. Engl. J. Med.*, **323**, 1517–1522.

LAURE, F., ROUZIOUX, C., VEBER, F., JACOMET, C., COURGNARD, V., BLANCHE, S., BURGARD, M., GRISCELLI, C. & BRECHOT, C. (1988). Detection of HIV 1 in infants and children by means of the polymerase chain reaction. *Lancet*, **ii**, 538–541.

LOWENFELS, A.B., MAISONNEUVE, P., CAVALLINI, G., AMMANN, R.W., LANKISCH, P.G., ANDERSEN, J.F., DIMAGNO, E.P., ANDREN-SANDBERG, A., DOMELLÖF, L. & THE INTERNATIONAL PANCREATITIS STUDY GROUP (1993). Pancreatitis and the risk of pancreatic cancer. *N. Engl. J. Med.*, **328**, 1433–1437.

SAIKI, R.K., GELFAND, D.H., STOFFEL, S., SCHARF, S.J., HIGUCHI, R., HORN, G.T., MULLIS, K.B. & ERLICH, H.A. (1988). Primer-directed amplification of DNA with a thermostable DNA polymerase. *Science*, **239**, 487–491.

SAMBROOK, J., FRITSCH, E.F. & MANIATIS, T. (1989). *Molecular Cloning*. Cold Spring Harbor Laboratory Press: Cold Spring Harbor, NY.

SIDRANSKY, D., TOKINO, T., HAMILTON, S., KINZLER, K.W., LEVIN, B., FROST, P. & VOGELSTEIN, B. (1992). Identification of ras oncogene mutations in the stool of patients with curable colorectal tumors. *Science*, **256**, 103-105.

TADA, M., OMATA, M. & OHTO, M. (1991). Clinical application of ras gene mutation for diagnosis of pancreatic adenocarcinoma. *Gastroenterology*, **100**, 233-238.

TADA, M., OMATA, M., KAWAI, S., SAISHO, H., OHTO, M., SAIKI, R.K. & SNINSKY, J.J. (1993). Detection of ras gene mutations in pancreatic juice and peripheral blood of patients with pancreatic adenocarcinoma. *Cancer Res.*, **53**, 2472-2474.

WARSAW, A.L. & DEL CASTILLO, C.F. (1992). Pancreatic carcinoma. *N. Engl. J. Med.*, **326**, 455-466.

YANAGISAWA, A., OTAKE, K., OHASHI, K., HORI, M., KITAGAWA, T., SUGANO, H. & KATO, Y. (1993). Frequent c-K-ras oncogene activation in mucous cell hyperplasias of pancreas suffering from chronic inflammation. *Cancer Res.*, **539**, 53-956.